# Vegetable Leaf Fermentation Improves Nutritional Quality of Sheep Feed, Enhancing Growth Performance and Intestinal Health

**DOI:** 10.3390/ani15223253

**Published:** 2025-11-10

**Authors:** Hongwei Xu, Kai Zhu, Yaodong Li, Huihao Wu, Rongxin Zang, Rui Zhou

**Affiliations:** 1 College of Life Science and Engineering, Northwest Minzu University, Lanzhou 730100, China; 2 Experimental Teaching Department, Northwest Minzu University, Lanzhou 730100, China; 3 State Key Laboratory for Zoonotic Diseases, Key Laboratory for Zoonosis Research of the Ministry of Education, Institute of Zoonosis, Changchun 130062, China

**Keywords:** fermentation, growth performance, intestinal, feed, sheep

## Abstract

The utilization of proper fermentation techniques is a widely recognized, efficacious approach in animal husbandry for enhancing the feed quality. However, research on vegetable waste, particularly that of roots, stems, leaves, fruits, and peels, has been rarely reported. To this end, the present study was carried out to examine the impact of vegetable leaf fermentation on growth performance, immune function, antioxidant levels, intestinal morphology, and microbial composition in sheep. Fifty-four male sheep (Oula) with an average age of 6 months and an average body weight of (21.53 ± 2.03) kg were randomly divided into three treatment groups, with six replicates each. The groups were fed with a basal diet (CON), 30% commercial fermented concentrate (CFC), and 30% vegetable leaf fermented concentrate (VFC). The results showed that compared to the CON group, both the commercial fermented concentrate and the vegetable leaf fermented concentrate improved the final weight (8.93%), average daily gain (30.67%), and dry matter intake of the sheep (1.62%). VFC increased the serum T-AOC (34.45%) and significantly increased the activities of serum and liver GSH-PX (10.95%). Meanwhile, the addition of vegetable leaf fermented concentrate increased the levels of serum IgA (63.21%), IgG (73.06%), and IgM (69.41%). VFC increased the villus height of the jejunum by 87.4% and the ileum by 185.5% and improved the villus height/crypt depth (V/C) ratio of the duodenum and ileum. CFC can also increase the villus height of the duodenum and jejunum, but has no effect on the morphology of the ileum. In addition to its other regulatory effects, VFC can further improve the richness and diversity of the rumen microbial community in sheep, with a notable enhancement in the relative abundance of key phyla, including *Bacteroidetes*, *Ascomycota*, *Zygomycota*, *Chytridiomycota*, and *Basidiomycota*. At the same time, the relative abundance of *Succinivibrio* was reduced. It can thus be concluded that the vegetable leaf fermented concentrate improves the growth performance and intestinal health of sheep.

## Simple Summary

This study examined the impact of vegetable leaf fermented concentrate (VFC) on sheep. Fifty-four 6-month-old Oula rams were divided into three groups: CON (basal diet), CFC (30% commercial fermented concentrate), and VFC (30% VFC). Results showed VFC better enhanced growth performance, antioxidant capacity, immunity, intestinal morphology, and rumen microbial diversity than CFC, while also addressing vegetable waste disposal issues.

## 1. Introduction

Vegetable waste is a general term for tissues that have lost their utilizable value during the production, processing, distribution, and consumption of vegetables. Among this waste, leaf waste mainly comes from old leaves of leafy vegetables and pure processed leaves. In contrast, whole vegetable residues contain multiple organs such as leaves, roots, and stems. Vegetable leaves feature a high water content and a low dry matter content, and are thus inconvenient for long-distance transportation and prone to rotting [1]. Currently, the main methods of disposal include underground burial and on-field dumping, which not only result in significant organic waste but also pose a huge threat to rural environmental safety. Feed, constituting approximately 70% of the overall production input expenses, matters considerably within the livestock industry [2]. In recent years, the prices of feed ingredients have been gradually increasing, thereby prompting researchers to explore alternative solutions to cope with the rising costs [3]. The application of plant protein sources, such as soybean meal, cottonseed meal, peanut meal, and rapeseed meal, to reduce animal protein in feed has been considered an effective cost-saving approach.

Solid-state fermentation is gaining recognition as a highly promising method for improving feed quality [4]. Recent studies have revealed that microorganisms involved in feed fermentation can produce beneficial compounds such as acetic acid, ethanol, acetaldehyde, and various aromatic acids [5]. Beyond enhancing the nutritional value, the fermentation process also generates bioactive factors, including biologically active peptides, which can inhibit the growth of harmful intestinal pathogens. The introduction of beneficial microorganisms from fermented feed into the intestinal tract establishes a dominant microbial population and thus fosters a competitive environment against detrimental microorganisms. Key strains utilized in fermentation include *Bacillus subtilis*, *Lactobacilli*, and yeasts, among others, with each strain offering distinct effects [5]. Notably, *Bacillus subtilis* supplementation during fermentation addresses deficiencies in endogenous digestive enzymes, while the resulting beneficial substances contribute to an improved gut microbial environment [6]. *Lactobacilli* are commonly employed for carbohydrate fermentation, converting carbohydrates into lactic acid and assisting in protein and fiber breakdown [7]. Recent research highlights the advantages of using mixed strains under optimal conditions, harnessing the full potential of each strain and achieving superior fermentation outcomes [8]. Extensive research has demonstrated that fermented feed improves growth performance, feed efficiency, digestibility, immunity, antioxidant capacity, and gut microbiota composition [9].

Vegetable leaves, as a type of household waste, tend to rapid spoilage and emit foul odors if not promptly and effectively managed, thus leading to significant environmental pollution [10]. The utilization of vegetable resources as animal feed can partially alleviate the shortage of feed ingredients in China, reduce environmental pollution, and promote sustainable resource utilization. Studies by Promkot et al. have shown that fermented cassava leaf feed can increase the number of rumen microorganisms in beef cattle and significantly improve their digestion and absorption of nutrients [11]. Research has indicated that feeding fermented *Broussonetia papyrifera* bark to meat sheep can reduce the feed conversion ratio [12]. However, existing research has focused on a narrow range of fermented plant materials, and there are critical gaps in the systematic study of fermented vegetable leaf feed: few studies have evaluated its comprehensive effects on sheep growth performance, nutritional digestibility, antioxidant, immune function, intestinal morphology, and rumen microbial composition, nor have they elucidated its potential regulatory mechanisms. Therefore, this study proposes the following hypothesis: exploring the use of probiotics to ferment vegetable waste to prepare fermented feed by clarifying its effects on the nutrient digestibility of sheep feed, promoting sheep growth performance, and enhancing the body’s antioxidant capacity and immune function, blood biochemical indicators, intestinal morphology, and rumen microbial community structure.

## 2. Materials and Methods

### 2.1. Probiotics and Culture

The microorganisms used in this study adhered to the established specifications outlined in the *Catalog of Feed Additives* (Announcement No. 2045) issued by the Ministry of Agriculture and Rural Affairs of China in 2013. This catalog provides guidelines and regulations for the use of microorganisms as feed additives, ensuring their safety and efficacy in animal nutrition. By following these specifications, the study complied with the regulatory standards set forth by the Ministry of Agriculture and Rural Affairs, thereby ensuring the suitability and reliability of the microorganisms employed in the fermentation process. *Bacillus subtilis* (CCTCC NO: 2021528), *Saccharomyces cerevisiae* (CICC NO: 1769), and *Lactobacillus plantarum* (CCTCC NO: M2021527) were all sourced from the College of Life Science and Engineering at Northwest Minzu University. The broccoli residue consists of broccoli leaves harvested in November, provided by Lanzhou Ruiyuan Agricultural Technology Co., Ltd. (Lanzhou, China). Priority is given to selecting processing by-products from broccoli-processing enterprises, such as outer old leaves and rhizome peels, or near-expiration and slightly damaged broccoli from supermarkets/farmers’ markets. For pretreatment, the collected broccoli residue is first spread out and sorted to remove rotten/molded parts, foreign objects like soil and stones, and thick and hard rhizomes (with a diameter exceeding 2 cm). Then, it is cleaned according to its source: processing by-products are sprayed with clean water at 0.2–0.3 MPa for 1–2 min. After cleaning, surface water is drained off, followed by air-drying in a cool and well-ventilated area for 8–12 h. Subsequently, the residue is crushed into particles with a size of 1–2 mm using a hammer mill. To prepare the vegetable leaf fermentation concentrate, a composite microbial agent consisting of *Bacillus subtilis*, *Saccharomyces cerevisiae*, and *Lactobacillus plantarum* was uniformly mixed in a mass ratio of 1:1:1. The mixture was then loaded into a fermentation bag equipped with a breathing valve. The fermentation bag was kept at room temperature and subjected to anaerobic fermentation for 5 days. The composition and nutritional profile of the vegetable leaf fermentation concentrate are presented in Table 1. The commercial fermentation concentrate was purchased from Xi’an Tieqi Leishi Feed Co., Ltd. (Xi’an, China), and the detailed nutritional components can be found in Table 1.

### 2.2. Experimental Design, Animals, and Diets

Fifty-four 6-month-old male Oula sheep were selected from Ruilin farm in Yongjing, Gansu Province, with an initial body weight of (21.53 ± 2.03) kg. After two weeks of adaptation, the sheep were randomly divided into three groups: the CON group (basal diet), the CFC group (30% commercial fermentation concentrate), and the VFC group (30% vegetable leaf fermentation concentrate), involving three replicates, with six sheep in each. Each group was raised in the same sheep house. During the pre-feeding period, the three groups of sheep were fed according to the original formula of the farm, with the experimental feed gradually added. They were given equal amounts of feed and drink at 08:00 and 18:00 every day. The composition and nutritional components of the basal diet followed the nutritional requirements set by the National Research Council (NRC) of the United States (National Research Council, 2012 [13]). Each column was equipped with RFID electronic devices to record the daily feeding data of each sheep. The weight of each sheep was measured at the beginning and end of the experimental period, and the average daily weight gain (ADG) was calculated based on the changes in weight.

### 2.3. Preparation and Sample Collection

At the conclusion of the 56th day of the experiment, all sheep were weighed, and three sheep were selected from each group for slaughter. The lambs were humanely slaughtered by severing the carotid arteries and jugular veins, following the halal procedure specified in Malaysian Standard MS1500:2009 [14]. Blood was drawn from the anterior vena cava, and the serum was separated through centrifugation. Subsequently, the lambs were eviscerated to collect rumen fluid, rumen, and liver tissue. The duodenum, jejunum, and ileum of each sheep were sectioned, and the chyme from the respective portions of the intestine was promptly frozen using liquid nitrogen. All samples were then stored in a refrigerator at −80 °C for preservation.

### 2.4. Apparent Digestibility Measurement

Prior to the conclusion of the feeding trial, sheep feces were collected twice daily for three consecutive days. After weighing, 100–200 g of feces was sampled, immediately mixed with 10% sulfuric acid (H_2_SO_4_) to preserve ammonia nitrogen, labeled, and stored at −80 °C for subsequent use. Upon thawing, the fecal samples were homogenized, dried at 65 °C for 72 h, and then allowed to equilibrate to ambient humidity at room temperature for 24 h. These fecal samples were used to determine the contents of dry matter (DM), crude protein (CP), and crude fat (EE). The apparent nutrient digestibility was evaluated using the AIA method, calculated by the following formula [13]:

Nutrient retention ratio (%) = ((WFI × NF) − (WEV × NE) × 100)/(WFI × NF)

WFI = weight of feed intake; NF = concentration of nutrient in feed; WEV = weight of total excreta voided; NE = concentration of nutrient in total excreta.

### 2.5. H&E Staining

Sheep tissue samples from the mid-duodenum, mid-jejunum, and mid-ileum were fixed in 4% paraformaldehyde for 48 h, then sectioned into 5 μm slices. After xylene dewaxing and ethanol hydration, hematoxylin and eosin (H&E) staining was used to examine tissue pathological changes.

### 2.6. Oxidative Stress Index Detection

According to the manufacturer’s instructions (Jiancheng, Nanjing, China), the serum of each group of sheep was tested using the corresponding detection reagent kit.

### 2.7. Determination of Blood Indices

For serum indices, total cholesterol (CHO), triglycerides (TG), total protein (TP), albumin (ALB), blood urea nitrogen (BUN), glucose (GLU), alanine aminotransferase (ALT), lactate dehydrogenase (LDH), alkaline phosphatase (ALP), and creatine kinase (CK) were determined using an automatic biochemical analyzer (Mindray BS-420, Shenzhen Mindray Bio-Medical Electronics Co., Ltd., Shenzhen, China).

### 2.8. Gut Microbiome Analysis

Herein, microbial genomic DNA was extracted from rumen contents using a QIAamp Fast DNA Stool Mini Kit (Qiagen Ltd., Hilden, Germany). The total DNA from the sample was extracted, and the V3-V4 hypervariable region was amplified using primer 338F (5-ACTCCTACGGGAGGCAGCA-3) and 806R (5-GGACTACHVGGGTWTCTAAT-3). Equimolar quantities of purified amplicons were combined and subjected to paired-end sequencing on the Illumina MiSeq PE300 platform/NovaSeq PE250 platform (Illumina, San Diego, CA, USA), following the established protocol of Majorbio Biopharmaceutical Technology Co., Ltd., Shanghai, China. The Uslust algorithm was used to cluster operational taxa (OTU) with a confidence threshold of 0.7. The Shannon index, Chao 1 index, and ace index were used to analyze the α diversity [13].

### 2.9. Statistical Analysis

All data were expressed as mean ± standard error of the mean (SEM) and analyzed using GraphPad Prism 8.0 software (GraphPad Software, San Diego, CA, USA). Duncan’s multiple range test was applied to compare the significance of differences among treatment groups, with *p* < 0.05 considered statistically significant. Meanwhile, normality and homogeneity of variance were tested using the Shapiro–Wilk and Levene tests, respectively. It should be noted that although the experiment was designed with 54 sheep allocated into three groups, only 3 sheep per group were selected for tissue sample collection.

## 3. Results

### 3.1. Growth Performance

The effect of adding fermented feed of vegetable leaves on growth performance is shown in Table 2. Compared with the CON group, the final body weight of sheep was increased in the CFC and the VFC groups (*p* < 0.05). The DMI of the starter in the CFC group and the VFC group was significantly higher than that of the CON group (*p* < 0.05). The daily gain of sheep was increased (*p* < 0.05), while the feed coefficient was decreased.

### 3.2. Nutrient Digestibility

According to Table 3, compared with the control group, the digestibility of DM, CP, EEm and NDF in the CFC group was significantly increased by 6.4%, 2.1%, 2.6%, and 5.5%, respectively (*p* < 0.05); the digestibility of DM, CP, and NDF in VFC group was significantly increased by 4.9%, 2.3%, and 7.5%, respectively (*p* < 0.05), while the digestibility of other nutrients had no significant change (*p* > 0.05).

### 3.3. Antioxidant Capacity

The effect of fermented feed of vegetable leaves on immune function is shown in Table 4. The oxidative stress indexes, such as total superoxide dismutase (T-SOD), were significantly improved (*p* < 0.05).

### 3.4. Immune Function

The effect of fermented feed of vegetable leaves on immune function is shown in Table 5. The VFC group of sheep had significantly higher levels of IgG, IgM, and IgA compared to the CON group and CFC group (*p* < 0.05), and though the difference was not statistically significant (*p* > 0.05), the commercial fermented feed group had higher values compared to the CON group.

### 3.5. Blood Biochemical Indexes

As shown in Table 6, the contents of triglyceride (TG), cholesterol (CHO), creatine kinase (CK), and alkaline phosphatase (ALP) in the VFC group were significantly lower than those in the CON group (*p* < 0.05), while the contents of total protein (TP), glucose (GLU), and urea nitrogen (BUN) in the VFC group were significantly higher than those in the CON group (*p* < 0.05). No significant difference was observed between the CFC group and the CON group (*p* > 0.05).

### 3.6. Intestinal Morphology

Table 7 presents the H&E staining results of the duodenum, jejunum, and ileum. Compared with the CON group, the villus length of duodenum, jejunum, and ileum, the depth of duodenum and ileum, and the V/C of duodenum in the CFC group were significantly increased (*p* < 0.05).

### 3.7. Intestinal Microbiological Analysis

**Figure 1:**
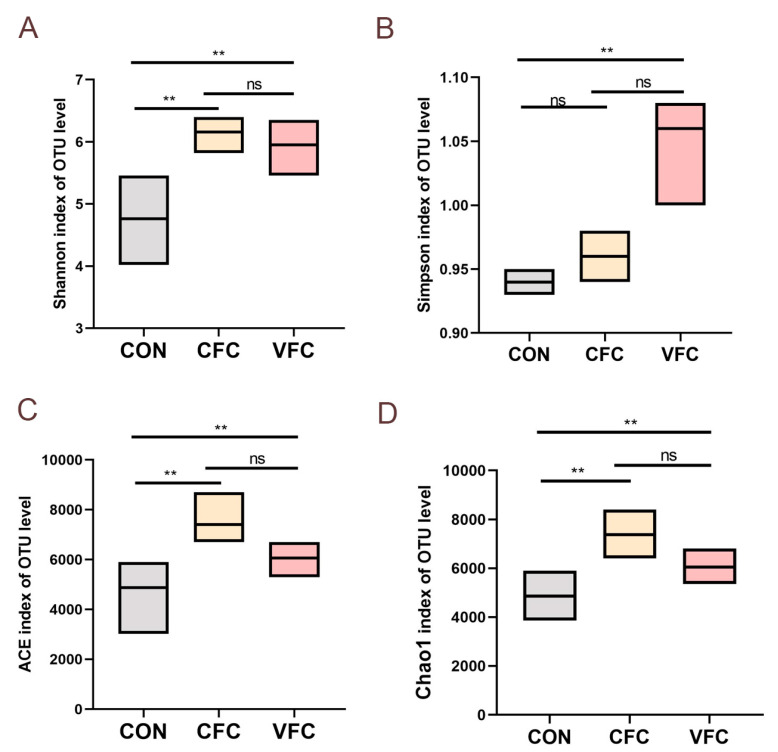
The richness and diversity index of species at 97% similarity level: Shannon (**A**), Ace index (**B**), Sobs index (**C**), and Chao index (**D**) (*n* = 6). SEM = Standard error of the mean. Asterisks indicate statistical differences between different groups: ** *p* < 0.01; ns: not significant.

**Figure 2:**
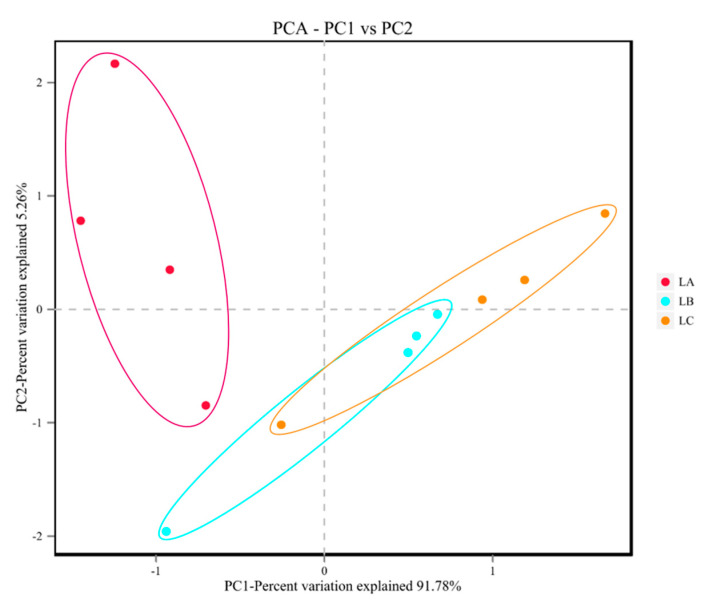
Species abundance PCoA analysis diagrams of horizontal Bray–Curtis algorithms. Group 1 = 30% commercial fermented concentrate (CFC); Group 2 = 30% vegetable leaf fermented concentrate (VFC) (*n* = 6). SEM = Standard error of the mean.

**Figure 3:**
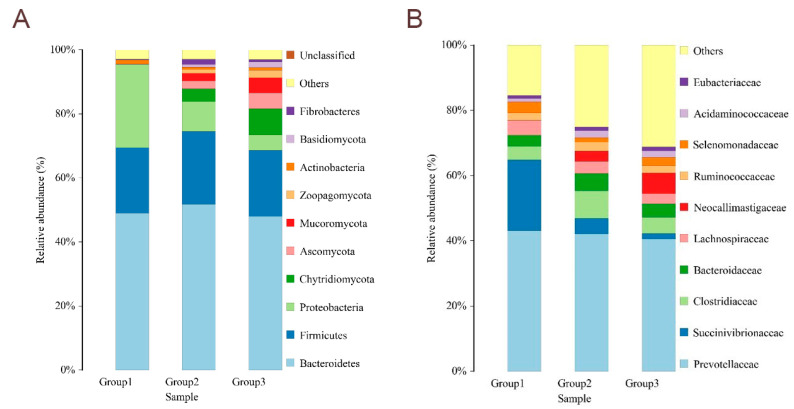
The abundance of intestinal microbiota at the phylum and class levels of sheep. (**A**) The abundance of intestinal microbiota composition at the phylum level. (**B**) The abundance of intestinal microbiota composition at the class level. Group 1 = 30% commercial fermented concentrate (CFC); Group 2 = 30% vegetable leaf fermented concentrate (VFC) (*n* = 6).

The CON group has 50 unique OTUs, the CFC group has 185 unique OTUs, and the VFC group has 57 unique OTUs. Shannon, Simpson, and Chao indices were presented for each group of samples to evaluate the alpha diversity of the sheep gut microbiota. Compared with the CON group, the addition of commercial premix improved the ACE, Sobs, and Chao indices (*p* < 0.05). Similarly, the addition of fermented feed from vegetable leaves increased the Shannon, ACE, and Chao indices (*p* < 0.05). It was thus determined that both commercial premix and fermented vegetable leaf feed increased rumen microbial diversity in sheep (Figure 1). Principal coordinate analysis (PCoA) and non-metric multidimensional scaling analysis (NMDS) were carried out to validate beta diversity. There was no significant overlap between the CON, CFC, and VFC groups in PCoA and NMDS when using the unweighted UniFrac distance. The PCoA analysis revealed a significant distinction in microbial community between CON and the other groups, with principal components PC1 and PC2 exhibiting differences of 18.73% and 13.35%, respectively (*p* < 0.05). All the findings indicated the considerable impact of introducing fermented feed into the sheep’s diet on their intestinal flora composition (Figure 2). In comparison to the CON group, *Bacteroidetes*, *Firmicutes*, *Proteobacteria*, *Chytridiomycota*, and *Ascomycota* are the most prevalent phyla at the phylum level. Herein, the *Proteobacteria* population was notably lower in CFC and VFC, *Chytridiomycota*, *Ascomycota*, and *Mucoromycota*. The presence of *Zoopagomycota* and *Basidiomycota* was notably higher (*p* < 0.05) (Figure 3A). Figure 3B displays the 0.01% composition of sheep rumen microbiota at the genus level. The presence of *Succinatimonas* and *Succinivibrio* was significantly lower (*p* < 0.05) in both the CFC and VFC groups when compared to the CON group. The presence of *Neocallimastix* and *Succiniclasticum* was notably higher in VFC (*p* < 0.05).

### 3.8. Functional Prediction

**Figure 4:**
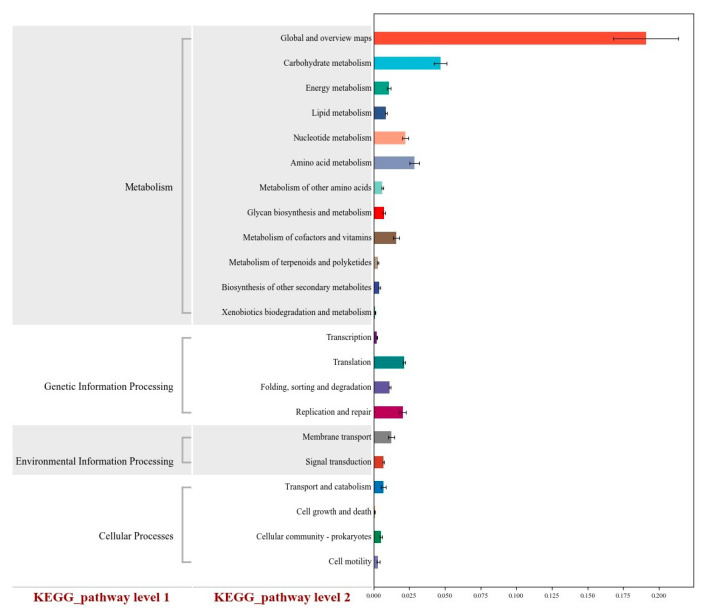
Statistical diagrams of functional genes related to KEGG metabolic pathway at secondary level (*n* = 6).

The abundance of functional categories based on KEGG level 2 was analyzed among the three groups (Figure 4). From the perspective of predicted results, significant differences in the predicted metagenomic potential were observed among the CON, CFC, and VFC groups across multiple KEGG pathways. In the predicted results related to metabolic functions, more than half of the single genes could be annotated to specific pathways. Compared with the CON group, the VFC group showed a significantly higher predicted enrichment level in ammonia carbohydrate metabolism, amino acid metabolism, and nucleotide metabolism pathways.

## 4. Discussion

Abundant bioactive substances in vegetable leaves are beneficial for improving the production performance of ruminants and have been extensively studied [15]. However, with the rise in national living standards, people’s demand for high-quality vegetables has also increased, so more discarded vegetables will be produced in the process of harvesting, storage, processing, loading and unloading, and transportation [16]. Vegetables, as a kind of daily garbage, easily rot, rapidly spoil, and produce odor if not effectively treated in time, thereby causing greater pollution to the environment [13]. Fermented feed enriches the content of probiotics, vitamins, organic acids, amino acids, polypeptides, enzymes, and growth-promoting factors. This feed-processing technology helps the digestion and absorption of nutrients by the host, thereby improving the growth performance of the animal [17,18]. Therefore, in this study, the mode of fermented feed was used to ferment the waste vegetable leaves, and the effect of fermented concentrated feed of vegetable leaves on sheep fattening was studied. The utilization rate of nutrient components in feed by animals determines the nutritive value of the feed. The better the degree of digestion and absorption of a specific nutrient by the animal’s gastrointestinal tract, the higher the digestibility of that nutrient will be. The results showed that compared with the CON group, the body weight and average daily gain of sheep were significantly increased, while the feed conversion ratio was significantly decreased by adding a vegetable-fermented feed. The above results indicate that fermented vegetable leaf concentrate can effectively improve the digestibility of nutrient substances in feed, thereby achieving the goal of enhancing the feed utilization rate.

The physiological function and metabolism of the body can be reflected by blood indicators involved in the physiological activities of the body through the circulatory system, and the blood components can also reflect the physiological and pathological changes in the animal body [19,20]. The health status of the body can be directly reflected by the content of triglycerides and cholesterol. Triglyceride is involved in the fat metabolism of the body. Generally speaking, the increase in triglycerides in the blood will promote the deposition of fat, leading to the occurrence of fatty liver and other diseases [21,22]. The decrease in triglyceride concentration indicates that the body’s utilization of fat increases and that the deposition of fat in the body decreases. Cholesterol is partly synthesized by the liver and partly provided by the diet [23]. Fat in the diet is digested and absorbed to form fatty acids, galactose, and glycerol, a small part of which is absorbed by the small intestine into the blood. In this case, the composition of lipids in the diet plays an important role in regulating serum triglyceride and cholesterol levels. Herein, the content of triglyceride and cholesterol was significantly reduced in the fermented concentrated feed of vegetable leaves, which improved the health status of sheep and promoted their growth and development. It was also found that the glucose and urea nitrogen contents increased significantly with the addition of the vegetable leaf fermentation concentrate [24]. The results showed that the addition of the concentrate of fermented vegetable leaves could improve the energy metabolism level of sheep, ensure the demand for rumen microorganisms, and promote the growth of animals. The possible reason for the increase in urea nitrogen content was that the protein content of the fermented concentrated feed of vegetable leaves was too high, so that the sheep’s body could not absorb it completely, indicating the necessity of further adjustment in the following experiment.

IgA, IgG, and IgM have been reported to be the key immune factors representing the immune level of sheep [25]. An important component of mammalian humoral immunity is immunoglobulin, which can enhance the phagocytosis of mononuclear macrophages, thereby inhibiting the reproduction of viruses and harmful microorganisms [26]. It was found that the levels of IgA, IgG, and IgM in the serum of sheep that were fed diets supplemented with vegetable-fermented feed were significantly increased. Through the above results, the addition of vegetable-fermented feed to the diet was found capable of improving the immune ability of sheep. In addition, oxidative stress is a common problem in sheep production, which seriously affects animal welfare and growth. A large number of studies have reported the existence of a variety of bioactive compounds in vegetable leaves, including bioactive substances and phenols [27]. These ingredients generally feature good physiological activities such as sterilization, anti-inflammation, anti-oxidation, anti-radiation, anti-cancer, and so on. T-AOC is a comprehensive index that can reflect the antioxidant capacity of the antioxidant defense system in vivo and in vitro [28]. Reactive oxygen species (ROS) scavenging enzymes such as T-SOD can reduce superoxide free radicals and hydrogen peroxide in piglets [29,30]. Based on the above results, it was found that the addition of vegetable-fermented feed in the diet could improve the antioxidant capacity of sheep by resisting oxidative stress. Intestinal morphology matters considerably in nutrient absorption, intestinal health, growth, and development of ruminants during the growing period. It has been reported that the height of intestinal villi is closely related to the nutritional efficiency of ruminants, and the higher the height of intestinal villi, the faster the rate of nutrient absorption. Studies have also shown that the depth of intestinal crypts reflects the degree of intestinal damage, and that intestinal epithelial tissue is the key to intestinal barrier function, which responds to inflammation through cytokines. A previous study has also indicated that fermented feed improves the performance of ruminants by tending to prolong the villus height and V:C ratio, which is consistent with the present study. Herein, it was found that a fermented vegetable leaf diet increased the villus height and V:C ratio in the intestines of sheep. Therefore, the protective effect of fermented vegetable leaf feed on intestinal barrier function resulted in better growth performance and feed efficiency in sheep [31].

The determination of rumen microbial community composition not only clarifies ruminant physiology but also helps to precisely manage animal nutrition and improve feed conversion. Intestinal flora is closely related to the host’s digestion and absorption of nutrients and immune response. For example, Wang et al. observed that roughage type had a significant effect on rumen microorganisms and metabolites, thereby altering growth performance. Studies have also shown that fermented feed can change the intestinal flora of animals [32]. However, the effects of fermented vegetable feed on intestinal microflora in sheep have been rarely investigated. To this end, a metagenomic sequencing approach was used to examine the rumen microbiome of sheep to assess any effects of the addition of vegetable-fermented feed to the diet. In this study, a decrease in ruminal microflora species, Shannon, Chao, and Ace indices was found in sheep-fed fermented diets, indicating an increase in ruminal microflora richness and diversity. In addition, the results of β-diversity showed that the gastrointestinal microbiota of the CFC and VFC groups were different from that of the CON group, which might also be related to the probiotic effect of fermented feed. *Bacteroidetes* and *Firmicutes* are associated with the digestion and decomposition of plant-derived diets by ruminants. *Bacteroidetes* favor the digestion of complex carbohydrates, and *Firmicutes* contribute to the digestion of fiber and cellulose [33]. In this study, the relative abundance of *Firmicutes* and *Bacteroidetes* was increased by the fermented feed of vegetable leaves. Similarly to the present results, previous studies have shown that the addition of fermented feed can increase the relative abundance of *Firmicutes* and *Bacteroidetes* in ruminants [34,35,36]. Meanwhile, *Prevotella* is the main bacterium that degrades starch in the rumen of ruminants [37]. *Clostridium butyricum* is the main *Clostridium* in the gastrointestinal tract, which, as a probiotic in the gastrointestinal tract, stimulates mucosal immune response by fermenting cellulose and producing butyric acid, thus inhibiting the growth of intestinal pathogens [38,39]. It was found that the relative abundance of *Prevotella* in the rumen of sheep was increased by the addition of fermented feed [40,41,42].

The KEGG functional prediction further confirms the metabolic mechanism underlying VFC’s beneficial effects. Compared with CON, the VFC group showed significantly higher enrichment in three key metabolic pathways: the carbohydrate metabolism pathway, the amino acid metabolism pathway, and the nucleotide metabolism pathway. The enrichment of the carbohydrate metabolism pathway activates the metabolic genes of core carbohydrate-degrading bacteria, optimizing the composition of VFAs and enhancing energy supply—this explains the higher ADG and lower FCR in the VFC group [43]. The enrichment of the amino acid metabolism pathway enhances transamination and deamination processes, improving the conversion efficiency of non-protein nitrogen to microbial protein, reducing nitrogen loss, and providing sufficient amino acids for the synthesis of serum immunoglobulins [44]. The enrichment of the nucleotide metabolism pathway provides raw materials for nucleic acid synthesis in beneficial microbes, supporting the increase in rumen microbial richness and diversity and consolidating the microbial foundation for nutrient utilization [43,44,45]. These three pathways form a “metabolic network” that links microbial function, nutrient metabolism, and animal growth, providing a clear molecular mechanism for the comprehensive beneficial effects of VFC. While this study demonstrates the significant value of VFC in sheep husbandry, it also has a notable limitation: the high BUN level in the VFC group indicates incomplete protein absorption, which requires further optimization of VFC’s nutrient composition in future research. Future studies should also explore the long-term effects of VFC on sheep (such as meat quality and reproductive performance) and identify the specific bioactive components in VFC that drive its antioxidant and immune effects. Additionally, the molecular mechanism by which VFC regulates rumen microbial communities (e.g., signaling interactions between microbes and the intestinal barrier) requires further investigation using multi-omics approaches.

## 5. Conclusions

In conclusion, replacing 30% of the basal diet with VFC significantly improves sheep growth performance, enhances antioxidant capacity and humoral immunity, optimizes intestinal morphology (especially ileal health) and rumen microbial diversity, and regulates blood biochemical indices to improve metabolic health—with effects that are generally superior to those of CFC. These benefits are driven by the synergy between VFC’s nutrient composition, bioactive substances, and regulatory effects on microbial metabolism. VFC not only provides a novel approach to valorizing vegetable waste but also offers a cost-effective alternative to commercial feed in sheep husbandry, holding broad application prospects in sustainable livestock production.

## Figures and Tables

**Table 1:** Experimental diet composition and nutrition level (air-dried basis).

Ingredients	CON ^1^	CFC ^2^	VFC ^3^
Corn straw (%)	33.70	36.6	43.40
Corn (%)	33.03	20.93	31.22
Concentrate supplement (%)	13.69	—	—
Commercial fermentation concentrate (%)	—	23.04	—
Vegetable leaf fermentation concentrate (%)	—	—	22.97
Wheat bran (%)	2.94	4.33	1.49
Soybean meal (%)	13.17	14.85	—
Stone powder (%)	0.17	0.08	—
Premix (%)	2.53	0.17	0.43
NaCl (%)	0.76	—	0.46
Total (%)	100.00	100.00	100.00
Nutrient levels			
DE (MJ/kg)	10.62	10.86	10.83
CP (%)	15.38	15.20	15.91
NDF (%)	2.87	17.96	17.08
ADF (%)	7.79	9.53	10.18
Ca (%)	0.60	0.46	0.62
TP (%)	0.37	0.39	0.49

^1^ The CON group (basal diet), ^2^ CFC group (30% commercial fermentation concentrate), and ^3^ VFC group (30% vegetable leaf fermentation concentrate, involving three replicates, with six sheep in each).

**Table 2:** Effects of fermented feed of vegetable leaves on growth performance of sheep (*n* = 6).

Item	Treatments			SEM	*p*-Value
	CON	CFC	VFC		
Initial weight (kg)	25.29 ± 3.04	26.24 ± 3.41	25.89 ± 2.62	2.40	0.546
Final weight (kg)	32.91 ± 2.80 ^b^	34.91 ± 3.66 ^ab^	35.85 ± 3.08 ^a^	1.70	0.043
Average daily gain, g/d	152.50 ± 23.00 ^c^	173.29 ± 20.94 ^b^	199.28 ± 25.92 ^a^	15.7	0.023
Average daily feed intake, g/d	1153.02 ± 14.22 ^b^	1153.72 ± 4.39 ^b^	1169.12 ± 13.12 ^a^	2.52	0.048

Feed conversion ratio = average daily feed intake/average daily gain. SEM = Standard error of the mean. ^a, b, c^ Different superscript letters in each indicator represent a significant difference (*p*-value < 0.05).

**Table 3:** Effects of vegetable leaf fermentation concentrate on nutrient digestibility of sheep (*n* = 6).

Item	Treatments			SEM	*p*-Value
	CON	CFC	VFC		
DM (%)	57.48 ± 0.42 ^b^	61.13 ± 0.27 ^a^	60.31 ± 1.02 ^a^	2.153	0.025
CP (%)	61.86 ± 2.70 ^b^	63.18 ± 1.80 ^a^	63.27 ± 2.36 ^a^	1.156	0.033
EE (%)	53.68 ± 0.55 ^b^	55.05 ± 0.43 ^a^	53.75 ± 0.87 ^b^	0.215	0.046
Ash (%)	46.40 ± 1.67	47.27 ± 1.30	46.55 ± 2.90	1.132	0.078
ADF (%)	45.47 ± 0.50	45.04 ± 0.93	46.46 ± 1.30	0.165	0.20
NDF (%)	52.49 ± 1.09 ^b^	55.39 ± 0.59 ^a^	56.45 ± 1.55 ^a^	2.103	0.045

DM = dry matter; CP = crude protein; EE = ether extract; NDF = neutral detergent fiber; ADF = acid detergent fiber. ^a, b^ Different superscript letters in each indicator represent a significant difference (*p*-value < 0.05). SEM = Standard error of the mean.

**Table 4:** Effects of fermented feed of vegetable leaves on antioxidant capacity of sheep (*n* = 6).

Item	Treatments			SEM	*p*-Value
	CON	CFC	VFC		
T-AOC, U/mL	18.08 ± 0.96	18.41 ± 0.99	24.31 ± 0.38 ^a^	0.456	0.042
T-SOD, U/mL	77.90 ± 5.50 ^b^	89.23 ± 7.78 ^ab^	107.03 ± 6.84 ^a^	0.565	0.026
CAT, U/mL	6.23 ± 0.48	6.66 ± 0.86	7.47 ± 0.82	0.781	0.056
GSH-Px, U/mL	831.69 ± 23.78	831.37 ± 13.38	919.67 ± 38.41 ^a^	2.563	0.046
MDA, nmol/mL	3.60 ± 0.10	3.56 ± 0.45	2.66 ± 0.36	0.876	0.075

SEM = Standard error of the mean. ^a, b^ Different superscript letters in each indicator represent a significant difference (*p*-value < 0.05).

**Table 5:** Effect of that fermented feed of vegetable leaves on the immune function of sheep (*n* = 6).

Item	Treatments			SEM	*p*-Value
Immune Function	CON	CFC	VFC		
IgA, mg/mL	19.60 ± 1.42 ^b^	20.62 ± 1.73 ^b^	31.99 ± 0.60 ^a^	0.519	0.035
IgG, mg/mL	23.20 ± 2.66 ^b^	25.38 ± 4.07 ^b^	40.15 ± 3.04 ^a^	0.316	0.025
IgM, mg/mL	10.66 ± 0.83 ^b^	11.17 ± 1.16 ^b^	18.06 ± 1.36 ^a^	3.01	0.015

IgA = immunoglobulin A; IgG = immunoglobulin G; IgM = immunoglobulin M; SEM = Standard error of the mean. ^a, b^ Different superscript letters in each indicator represent a significant difference (*p*-value < 0.05).

**Table 6:** Effects of different diets on blood biochemical indexes in sheep (*n* = 6).

Item	Treatments			SEM	*p*-Value
	CON	CFC	VFC		
TP (g/L)	61.97 ± 0.70 ^b^	64.50 ± 1.97 ^ab^	66.50 ± 0.85 ^a^	3.153	0.013
ALB (g/L)	29.633 ± 1.22	30.07 ± 1.20	33.53 ± 1.22	0.456	0.085
BUN (mmol/L)	1.63 ± 0.37 ^b^	1.85 ± 0.33 ^b^	6.62 ± 1.19 ^a^	3.415	0.001
CK(U/L)	263.67 ± 9.61 ^a^	257.33 ± 10.79 ^ab^	211.33 ± 16.17 ^b^	15.15	0.023
DAO (U/L)	10.01 ± 0.20	10.11 ± 0.62	10.08 ± 0.52	0.165	0.15
ALP (U/L)	510.67 ± 8.08 ^a^	508.33 ± 5.69 ^a^	425.33 ± 18.01 ^b^	10.16	0.014
CHO (mmol/L)	1.53 ± 0.11 ^ab^	1.48 ± 0.02 ^a^	1.17 ± 0.04 ^b^	0.125	0.021
TG (mmol/L)	0.39 ± 0.01 ^a^	0.41 ± 0.06 ^ab^	0.25 ± 0.02 ^b^	0.054	0.016
ALT (U/L)	24.67 ± 0.57	25.00 ± 1.00	25.00 ± 1.00	0.578	0.685
LDH (U/L)	4.67 ± 0.27 ^b^	5.30 ± 0.24 ^b^	6.11 ± 0.19 ^a^	1.124	0.047

SEM = Standard error of the mean. ^a, b^ Different superscript letters in each indicator represent a significant difference (*p*-value < 0.05).

**Table 7:** Effects of vegetable leaf fermented feed on intestinal morphology of sheep (*n* = 6).

Item	Treatments			SEM	*p*-Value
	CON	CFC	VFC		
Villus height (V)					
Duodenum, mm	782.80 ± 122.63 ^b^	1040.93 ± 113.84 ^a^	824.27 ± 115.52 ^ab^	2.045	0.016
Jejunum, mm	614.03 ± 96.84 ^c^	909.20 ± 22.31 ^b^	1150.73 ± 119.53 ^a^	3.078	0.001
Ileum, mm	283.67 ± 24.42 ^b^	682.83 ± 134.53 ^a^	809.87 ± 49.64 ^a^	0.468	0.001
Crypt depth (C)					
Duodenum, mm	386.13 ± 41.09 ^b^	494.83 ± 51.86 ^a^	363.37 ± 25.11 ^b^	0.814	0.032
Jejunum, mm	396.30 ± 32.33 ^b^	464.60 ± 14.67 ^b^	613.33 ± 66.51 ^a^	0.054	0.001
Ileum, mm	169.07 ± 7.70 ^c^	285.13 ± 11.18 ^b^	372.70 ± 35.78 ^a^	0.785	0.001
V:C ratio					
Duodenum, mm/mm	2.03 ± 0.22	2.10 ± 0.10	2.27 ± 0.32	0.695	0.09
Jejunum, mm	1.55 ± 0.20	1.96 ± 0.11	1.90 ± 0.41	0.478	0.06
Ileum, mm	1.68 ± 0.19 ^b^	2.40 ± 0.48 ^a^	2.19 ± 0.33 ^ab^	0.896	0.042

SEM = Standard error of the mean. ^a, b, c^ Different superscript letters in each indicator represent a significant difference (*p*-value < 0.05).

## Data Availability

All data generated during the current study are included in this article. The sequencing data in the present study are available in the NCBI Sequence Read Archive (SRA) repository under the accession number PRJNA1049638.
